# Factors Associated with Increased or Decreased Stress Level in French Children during the First COVID-19 Lockdown

**DOI:** 10.3390/ijerph20054667

**Published:** 2023-03-06

**Authors:** Juliette Faucher, Nagham Khanafer, Nicolas Chauliac, Aziz Essadek, Perrine Galia, Elise Mamimoue, Marie-Laure Leroux, Marie-Pierre Pollet, Françoise Imler-Weber, Yves Gillet, Pierre Fourneret, Pauline Espi

**Affiliations:** 1 Service Psychopathologie du Développement de l’Enfant et de l’Adolescent, Hôpital Femme Mère Enfant, Hospices Civils de Lyon, 69500 Bron, France; 2 Service d’Hygiène, Épidémiologie et Prévention, Hôpital Edouard Herriot, Hospices Civils de Lyon, 69003 Lyon, France; 3 Regional Center for Psychotraumatism, Hôpital Edouard Herriot, Hospices Civils de Lyon, 69003 Lyon, France; 4 Interpsy EA4432, University of Lorraine, 54000 Nancy, France; 5 Academy of Lyon, Ministry of National Education, 75007 Paris, France; 6 Department of Pediatric Emergency, Hôpital Femme Mère Enfant, Hospices Civils de Lyon, 69500 Bron, France

**Keywords:** COVID-19, lockdown, stress, anxiety symptoms, family relationship, academic pressure

## Abstract

In spring 2020, governments of many countries implemented lockdown measures to prevent the spread of the COVID-19 pandemic. Worldwide, the pandemic forced about 1.5 billion children to stay at home for several weeks and to experience homeschooling. The objective of this study was to assess the variation in stress levels and associated factors in school-aged children in France during the first COVID-19 lockdown. A cross-sectional study using an online questionnaire was designed by an interdisciplinary team involving hospital child psychiatrists and school doctors. Between 15 June and 15 July 2020, Educational Academy of Lyon (France) invited the parents of school-aged children to participate in this survey. The first part of the questionnaire concerned the children with data on lockdown conditions, socio-demographic data, daily rhythms (eating and sleeping), perceived stress variations, and feelings. The second part assessed parental perspectives on their child’s psychological state and use of the mental health care system. Multivariate logistic regression was performed to identify factors associated with stress variation (increased or decreased). A total of 7218 questionnaires were fully completed by children from elementary school to high school with a balanced sex ratio. In summary, 29% of children reported a higher stress level during the lockdown, 34% reported a lower stress level, and 37% reported no stress variation in the usual situation prior to COVID-19. Parents were most often able to identify signs of increased stress levels in their children. The most influential factors in the variation of stress for children were academic pressure, family relationships, and fear of being infected or infecting a family member with SARS-CoV-2. Our study underlines the high impact of school attendance stressors on children in usual conditions and encourages vigilance for children whose stress levels have decreased during the lockdown but who may have increased difficulty re-exposing themselves upon deconfinement.

## 1. Introduction

On 11 March 2020, the World Health Organization (WHO) declared a worldwide pandemic due to the severe acute respiratory syndrome coronavirus 2 (SARS-CoV-2), causing coronavirus disease 2019 (COVID-19) [1]. To minimize the spread of SARS-CoV-2, countries all around the world established various measures such as hand hygiene, mask wearing, and social distancing. One of the most extreme ways to achieve social distancing was the lockdown of the entire population. Not exactly knowing at the time whether children were at risk of infection or were at risk to highly transmit the virus, countries chose to close schools and all places where children can gather. According to the United Nations Educational Scientific and Cultural Organization (UNESCO), schools have been suspended nationwide in 188 countries. In April 2020, more than 1.2 billion children and adolescents worldwide found themselves isolated at home, causing extensive disruption to their lives [2].

During previous epidemics, due to severe acute respiratory syndrome coronavirus (SARS-CoV), Influenza A virus (H1N1), or Ebola virus, there was an increase in child poverty and conflicts at home, a decrease in food intake, and a reduction in parents’ employment levels [3]. During these epidemics, school interruption negatively impacted school learning and child protection, while increasing the risk of permanent dropout [3]. Some studies focused on children’s mental health reported increased rates of depressive symptoms, and higher risk of acute stress disorder, adjustment disorder, and grief [4,5]. A study of American families exposed to the H1N1 and SARS-CoV viruses reported PTSD in 30% of children exposed to quarantine measures [4]. The adult population presented more frequent anxiety and depressive and post-traumatic symptoms, and parents’ psychological distress was linked to poorer well-being in children [4,6].

General lockdowns and closings have been associated with reduced social life and physical activity, changes in routine, risk of sleep disturbances, exposure to discord at home, excessive screen use, and unhealthy diet in children and adolescents [7]. Consistent evidence shows that the structured environment of weekdays may help reduce stress in children by bringing them a feeling of safety. It also has a positive impact on their health by regulating obesogenic behaviors [8].

During the COVID-19 pandemic, children and adolescents experienced a prolonged state of physical isolation from their peers, teachers, extended families, and community networks. Social isolation and feelings of loneliness can lead to the development of anxiety and depression symptoms [9,10,11]. An additional stress factor on the pediatric population was the initial uncertainty about children’s contagiousness and their responsibility for viral transmission [12].

The first studies about the psychopathological impacts of the lockdown associated with the COVID-19 pandemic reported an increased prevalence of anxiety and depression symptoms in children and adolescents. Some suggest that the prevalence of depression might have doubled since the start of the pandemic [13], with an increase in rates of anxiety symptoms of a wide range from 8% to 74% [14]. A drop in reports of maltreatment situations was also observed all around the world, whereas an exact evaluation of maltreatment true incidence is lacking [15]. Other studies found that in populations where the academic burden is high, the level of anxiety might decrease compared to before the lockdown [16]. This also happened when children and adolescents received good support within the family, were engaged in activities together, and were able to communicate and share their emotions [17,18].

To obtain data to better understand the complexity of the psychological impact of the lockdown associated with the COVID-19 pandemic on children and adolescents, we decided to study the variation in stress levels in a large population of French school-aged children. In contrast to other studies in the literature, we decided to investigate globally the impact of the lockdown on children, irrespectively of their potential negative or positive aspects. We looked for factors associated with an increase or decrease in their stress levels to identify possible subgroups of children at risk and to formulate public health recommendations. We studied risk factors related to socio-demographic aspects (gender, academic level, parents’ socio-professional categories, and urban/rural lifestyle), children’s activities and daily rhythms (eating and sleeping), negative and positive aspects of the lockdown as perceived by the children, their fears related to the illness, their worries and expectations about returning to school, and parents’ perceptions of their child’s psychological state.

## 2. Materials and Methods

### 2.1. Study Objectives and Procedures

The PSICOcs (Psychological Impact of COVID-19 Epidemic on School Aged Children) study was designed by a team from two hospitals of Lyon University Hospital (Hôpital Femme Mère Enfant and the Regional Center for Psychotrauma), in association with the Academy of Lyon. PSICOcs is a cross-sectional study based on an online questionnaire. The rectorate wrote an information letter about the study to all school directors in the Lyon Academy. Between the 15 and 22 June 2020, school directors relayed the information about PSICOcs study and then the questionnaire via a digital link, sent by email to parents of school-age children. The same link allowed parents and children to complete the questionnaire, with instructions, so that participants would know which questions were intended for children or parents. The responses were collected up to the 15 July 2020. To reduce memory bias, we started the study as close as possible to the end of the lockdown and school reopening, and the study ended at the start of the summer school holidays.

**Figure 1:**
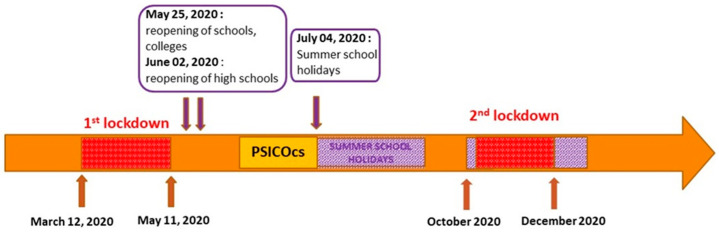
Timeline of the PSICOcs study.

The participation was anonymous, and the answers were stored on the digital platform LimeSurvey, an online hosting site. Details of the procedure period are described in “Study Timeline” (Figure 1).

### 2.2. Study Population

The study population was the 500,000 children and adolescents aged 6 to 18 years registered in the Lyon Academy (INSEE, 2020). This academy is made up of three departments (Ain, Loire, and Rhône), each of which has its own referring school physician in conjunction with the school directors. There are 2664 schools in the Lyon Academy.

### 2.3. Material

A global questionnaire was designed, including a part addressed to children and a part addressed to parents. It consisted of a total of 32 questions, divided as follows:

- Parental consent to the questionnaire (1 question)
- Sociodemographic characteristics: child’s gender, child’s academic level, type of school (public or private), parent’s professions, and city of residence (5 questions)
- Living context during confinement: the presence of family members at home, urban or rural lifestyle, activities practiced, modification of daily rhythms (eating and sleeping), screen consumption time (for schoolwork and leisure time, respectively) (7 questions)
- Negative and positive perceived aspects of confinement with Lickert-type responses: level of stress during confinement compared to the usual situation (“much more stressed”, “a little more stressed”, “stressed as usual”, “a little less stressed”, and “much less stressed”), worries about the disease, potential positive aspects of confinement, and concerns about the confinement (7 questions)
- Worries and expectations about returning to school with Likert-type scales (anxiety of separation from the family, fears about COVID-19 and academic learnings, hopes concerning the resumption of activities and social interactions, and the degree of acceptance of sanitary measures at school) (6 questions)
- An estimate of the child’s temperament based on the “AES Approach to temperament” [19], with the inclusion of three items from each dimension (emotionality, activity, and shyness) to calculate an emotional score
- A section addressed to parents with Lickert-type responses: level of concern about the disease, level of concern about school returning, potential psychological follow-up of their child prior to COVID-19, worries about the psychological state of their child during confinement, use of the health care system during the confinement, and confidence in governmental decisions (6 questions)

We tried to simplify the questionnaire as much as possible to collect only the relevant elements and to increase the chance of completion by a large number of children. We adapted vocabulary and grammar to 6-year-old children.

The global context of the study and the objectives of the questionnaire were presented in the introduction on the first page. At the end of the questionnaire, we delivered some psychoeducation advice based on current recommendations about children’s mental health.

Data on the socio-professional categories of the parents are presented as used by the French National Institute for Statistics and Economic Studies (INSEE). Categories of occupations were organized as follows (according to INSEE classification):

- Craftsmen, traders, farmers, and entrepreneurs
- Executives and higher intellectual professions
- Employees and workers
- Intermediary profession
- Without professional activity

### 2.4. Statistical Analysis

Statistical analyzes were conducted for fully completed questionnaires (i.e., completed up to the last page). Participants were categorized into three groups, based on their responses concerning the variation of stress levels during the lockdown, compared to before lockdown: a group of school-aged children who felt more stressed during the lockdown, a group of children feeling less stressed, and a group of children who did not report any variation in their general level of stress. Summary tables (descriptive statistics) are provided for all baseline variables as appropriate. Continuous variables were summarized as means (min-max) or median (IQR), and categorical variables were described by frequency and percentage. The normality was assessed using a Shapiro–Wilk test, categorical variables were compared using Fisher or Chi-square tests, and continuous variables were compared using Student or Mann-Whitney tests.

Univariate and multivariate logistic regression models were used to identify factors associated with increased or decreased stress in participating children. The variables with a *p*-value ≤ 20% in the univariate analysis or considered clinically important were retained for the multivariate analysis by a stepwise ascending method. The interactions between the variables retained in the multivariate analysis were tested. The different models were then compared using the likelihood ratio test to select the goodness of fit of the model. Results were expressed as odds ratio (OR) and 95% confidence intervals (CI 95%). All *p*-values were two-tailed. *p*  <  0.05 were considered statistically significant.

Statistical analyses were performed with the IBM SPSS Statistics for Windows, Version 19.0. Armonk, NY, USA: IBM Corp. and R statistical software (R Foundation for Statistical Computing, Vienna, Austria. URL: http://www.R-project.org/ (accessed on 21 February 2023)).

### 2.5. Ethics Consideration

This study was approved by the Ethics Committee for Biomedical Research of the Hospices Civils de Lyon and registered in ClinicalTrials.gov under the reference NCT04475484. This work was supported by the National Education and locally by the Rectorate of Lyon. Parental consent was obtained for all participants. Data were anonymized and stored on a secure server. None of the participants received financial compensation.

## 3. Results

### 3.1. Participants and Demographic Characteristics

The web-based questionnaire was accessed 15,273 times over a four-week period and 7314 completed questionnaires were returned (47.9% completion rate). Considering a population of 500,000 children in the Academy of Lyon, the response rate is approximately 1.4%. After excluding questionnaires completed without parental consent, a total of 7218 school-aged children and their parents were included in the analysis.

Of the included children and adolescents, 52.3% were girls, and 46.3% were boys. At the time of participation, 15.4% were in high school, 43.5% in middle school, and 39.5% in elementary school. The majority of participants (70.5%) lived in urban areas, mainly in a house (80.4%) (with a private garden for 78.6% of them), while 17.7% lived in an apartment. Most parents worked as employees or workers, or in an intermediate profession. Less than 15% were executives, company managers, or in a higher intellectual profession. Less than 10% were unemployed or retired. During the lockdown, 70.0% of children lived with siblings, 87.0% lived with their mother, and 66.9% with their father. For 7.4% of them, a stepfather or stepmother was present, and a grandparent was present in 3.5% of households. At home, 39.4% had a pet. The complete demographic characteristics, as well as the descriptive data set, are presented in Supplementary Materials.

### 3.2. Primary Outcome

Approximately 1/3 (33.3%) of the children reported a decrease in their stress level during the lockdown compared with the usual situation (S-population), approximately 1/3 (28.1%) reported an increase in stress (S+ population), and approximately 1/3 (36.7%) reported no change (S0 population), with 1.9% missing data.

### 3.3. Univariate Analysis

We compared the “increased stress level” (S+) group and the “decreased stress level” (S-) group with the “as usual stress” (S0) group using univariate analyses. All results are presented in Table 1.

Several factors were significantly associated with a higher level of stress during the lockdown, such as lacking guidance in academic learning by a professor (OR = 2.69, 95% CI [2.26; 3.20]), feeling lonely (OR = 2.41, 95% CI [2.09; 2.78], feeling less motivated (OR = 1.92, 95% CI [1.70; 2.16]) or less efficient (OR = 1.87, 95% CI [1.66; 2.12]) in their schoolwork. Perceiving more conflicts at home was also positively associated with feeling more stressed (OR = 2.00, 95% CI [1.72; 2.32]).

A lower level of perceived stress during the lockdown was significantly associated with being in middle school (OR = 1.46, 95% CI [1.29; 1.65]) or in high school (OR = 1.61, 95% CI [1.36; 1.90]) compared to being in primary school, as was feeling less school pressure during lockdown (OR = 2.63, 95% CI (2.35; 2.95]).

Some variables were found to be significantly associated both with S- and S+ like being regularly followed by a mental healthcare professional, or spending more than 4 h on screens for homework with a dose-response relationship for screen time. Worrying about leaving the family when going back to school was also associated with both groups.

### 3.4. Multivariate Analysis

#### 3.4.1. Factors Associated with a Higher Stress Level during Lockdown

In multivariate logistic regression analysis, factors positively associated with feeling more stressed during the lockdown included spending more than 4 h on screens for school purposes (OR = 1.58, 95% CI [1.18; 2.12]), feeling less motivated for schoolwork (OR = 1.56, 95% CI [1.34; 1.82]), and worrying about school learning (OR = 2.34, 95% CI [1.84; 2.96]). Fear of getting ill (OR = 2.12, 95% CI [1.61; 2.80]) or fear of a family member getting ill (OR = 1.55, 95% CI [1.18; 2.05]) was also significantly associated with S+, as was feeling lonely (OR = 1.31, 95% CI [1.09; 1.58]), or witnessing more tension at home (OR = 1.24, 95% CI [1.02; 1.51]).

Parents’ perception of their child being more withdrawn, disruptive, worried, sad, or suffering than before the lockdown is positively associated with their child reporting feeling more stressed.

Multivariate analyses are presented in Table 1.

**Figure 2:**
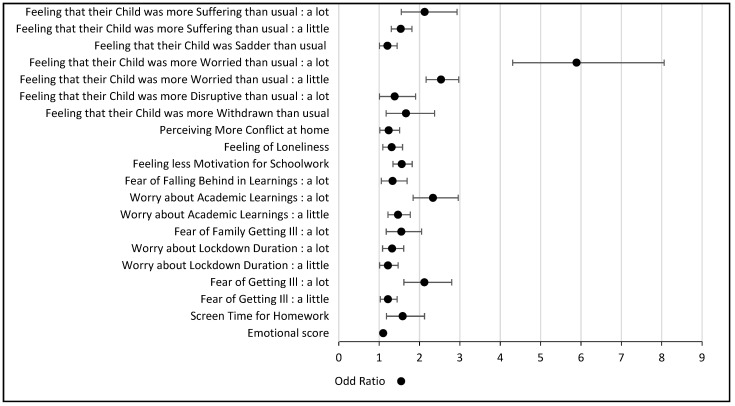
Risk factors significantly associated with S+.

Risk factors are independently associated with a higher stress level during the lockdown, and their importance is represented in Figure 2.

#### 3.4.2. Factors Associated with a Lower Stress Level during Lockdown

**Figure 3:**
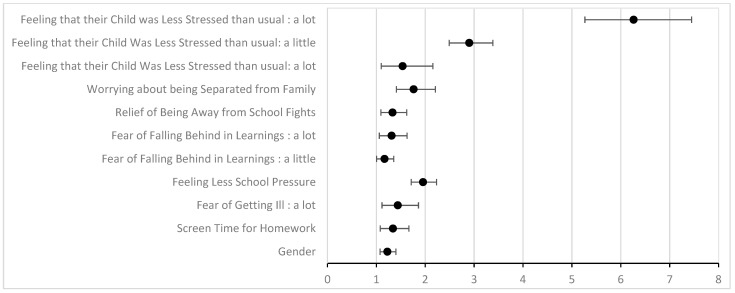
Risk factors significantly associated with S-.

Factors significantly associated with feeling less stressed during the lockdown (Table 1 and Figure 3) were feeling less academic pressure (OR = 1.96, 95% CI [1.71; 2.23]), enjoying being away from school fights or bullying (OR = 1.33, 95% CI [1.09; 1.62]), spending between 2 and 4 h on screens for school purposes (OR = 1. 34, 95% CI [1.08; 1.67]), fear of falling behind academically (OR = 1.31, 95% CI [1.06; 1.63]), fear of being separated from family when going back to school (OR = 1.76, 95% CI [1.41; 2.21]), and fear of getting sick (OR = 1.44, 95% CI [1.12; 1.86]).

Parent’s perception of their child being less stressed is significantly associated with the child reporting less stressed (OR = 6.26, 95% CI [5.27; 7.45]).

Risk factors are independently associated with a lower stress level during the lockdown, and their importance are represented by Figure 3.

## 4. Discussion

To the best of our knowledge, this is the largest study regarding the psychological wellbeing of school-aged children in France during the first lockdown. Our population sample is comparable with the general French population in terms of gender proportion, presence of siblings, and parent’s occupations, but differs concerning the proportion of school grade levels and lifestyles. Our study includes a large proportion of children who report living in a house with a garden, which is likely to be a rural lifestyle. It may also indicate a higher socio-economic level of the responders compared to the general population.

### 4.1. Variation of Stress Levels in Children during Confinement Compared to Usual Situation (Prior to COVID-19)

We found that two third of participating children and adolescents adapted well enough to the situation that they felt no variation of stress or felt even better under lockdown situations. This differs from most of the literature, which reports a general deterioration in child and adolescent mental health due to the COVID-19 crisis and the different sanitary measures put in place, especially lockdowns and school closures [20]. As it has then been confirmed by the reports on child and adolescent suicidal behaviors since the second semester of 2020 [21], these data must not be overlooked. However, by looking only for negative impacts and using only psychopathology scales, studies may not identify the potential positive aspects of lockdown as experienced by children, as well as coping strategies [22]. Other studies that explored both negative and potentially positive impacts of the lockdown on children have found more contrasted data [23]. In the same way, our results point out that a certain proportion of school-aged children (34%) felt less stressed in lockdown conditions compared to usual circumstances. This is different from the COVID-19 literature, which mostly examined risk factors for mental health deterioration. We can hypothesize that this research methodology was related to the fact that an increase in anxiety and depression symptoms in the global population was the main expectation and concern at the beginning of the pandemic.

### 4.2. Decrease in Stress Levels during the Lockdown

To understand our results, in particular, among the third of the children who reported a reduction in their stress level during the lockdown, we can propose several hypotheses. The first hypothesis can be based on the impact of the family atmosphere during these times. Several studies have highlighted the importance of good family relationships in buffering the effects of the crisis [14,24]. Others went further, suggesting that a more intense family life could lead to an alleviation of stress and mental health problems experienced by children before the crisis [23,25]. Some parents used the closure as an opportunity to spend more time with their children, helping them with their schoolwork, playing with them, or engaging them in different activities [26]. This contributed to a better understanding of the children and improved their relationship [27].

Second, the difficulties faced by school-aged children before the crisis and their sense of isolation need to be explored. The question of school academic and social pressure is highlighted in our study, with only 55% of the study population who were glad to go back to school. During the lockdown, up to 15% of our population were glad to be away from other school kids, and 14% reported feeling better away from school fights or bullying. The impact of social pressure but mostly bullying at school has now been well documented and known to be a risk factor for school refusal a long time before COVID-19 [28]. Up to 38% of our population felt relieved about the alleviated academic pressure, which was positively associated with lower stress levels (OR = 1.96) and negatively associated with a high level of stress as reported in other studies. In fact, a Korean study reported that 21.4% of the school-aged population feels less stressed during lockdown [16].

Thirdly, academic-related worries can impact stress level in children in different ways and was found significantly associated with a higher or lower level of stress, with a stronger association with feeling more stressed. Feeling intensively worried about academic learning was found to be significantly associated with a higher stress level, which is also consistent with the literature [29]. The same phenomenon of both increased and decreased stress for the same risk factor was also found for illness-related concerns. We can hypothesize that some children who were particularly worried about getting sick were highly reassured by the lockdown and therefore experienced a decrease in their stress level, while others experienced an overall increase in their stress level despite the measures or even in relation to the protective measures. These results might represent the heterogeneity of our sample population, and the complexity of individual reactions to the same stressor. This could also be related to the presence of confounding factors not investigated in our study.

### 4.3. Increase in Stress Level during the Lockdown

Most of the other factors found positively associated with a higher level of stress are consistent with the current COVID-19 literature. In our study, up to 17% of children and adolescents reported more conflicts at home, which was positively associated with a higher level of stress. Being forcibly lockdown with family can also have its downside. Several studies have reported elevated risk factors for family conflicts such as living in small spaces, having difficult relationships before the lockdown, enduring financial or job loss, or having to work from home while caring for children [6,25,30].

A high emotional score (as evaluated by the temperament items) was also positively associated with a higher level of stress, which is coherent with the definition given by Plomin and Buss who stated that “compared to unemotional people, emotional people become more distressed when confronted with emotion-laden stimuli” [19]. A study on the COVID-19 outbreak impact on families also reported that adolescents with limited access to emotion regulation strategies were twice as likely to experience increased psychological stress, identifying difficulties in emotion regulation as risk factors of increased psychological stress [29].

Another factor associated with higher stress levels was feeling of loneliness. Several studies have reported a strong association between the feeling of loneliness and psychiatric symptoms of depression and anxiety, with the duration of this feeling being a strong predictor of mental health issues up to 9 years later [31].

The fear to transmit the disease and therefore to be responsible for the illness (even severe one with the risk of death) of their relatives was also frequently reported by the children feeling more stressed during the lockdown. It is quite unusual that children worry about their own responsibility during the global crisis and maybe a specific aspect of these pandemics. To our knowledge, this point was not evaluated in other studies but should be considered during the global evaluation of the consequences of lockdowns. Routine modification has not been found to be relevant in our study population, even though it has been linked in other studies with the deterioration of well-being in child’ populations. Lack of physical activity, altered sleeping patterns, and unhealthy or increased food intake were considered major risk factors for higher anxiety symptoms in the literature [32]. The absence of impact of dysregulation in sleep rhythm in our study could be explained by a return to sleeping patterns not synchronized to social constraints. Especially during adolescence, sleeping patterns are known to be modified by school rhythm [20].

### 4.4. Parents’ Perceptions of Their Child’s Psychological State

Children’s stress levels and parental perceptions about their psychological state were in good adequation. Parents perceiving their child being more disruptive, more shut down, more worried, and suffering during lockdown was associated with their child reporting feeling more stressed. They were also able to identify positive changes such as a diminution in their child’s stress. Few articles focus on parent–child agreement about children’s and adolescents’ stress levels or mental health. A review from 1991 suggests that the responses might differ consistently [33], while a more recent study tempers this, finding that parents might be good evaluators of externalised disorder but might be less aware of internal disorders in their children, while children and adolescents can themselves report these disorders, concluding on the importance of asking both parents and children [34]. In our study, parents were able to evaluate stress, disruption, and suffering, but sadness and withdrawal seemed less evident for them to recognize in their child.

### 4.5. Limitations of Our Study

As stated in the CHERRIES checklist for online surveys, our survey, similar to many online surveys, had a low participation rate [35]. The population sample was not what we can call an “internet population” as parents were emailed by the regional school administration and did not go on their own to a website looking for this kind of information, but there was a “volunteer bias” as nothing made it mandatory and no kind of compensation was offered.

The fact that some factors were associated with both more stressed and less stressed groups might appear contradictory. It could be explained by confounding factors that were not assessed in our questionnaire. Several factors that we did not explore have been relevant in other studies, such as financial hardship and food insecurity [4,36]. Another possible confounder would be the resilience capacity of these families and children. Resilience is the process and outcome of successfully adapting to difficult or challenging life experiences, especially through mental, emotional, and behavioural flexibility and adjustment to external and internal demands [37]. It is a protective factor against stress or other mental health symptoms when facing extreme events such as this pandemic and is strictly connected to children’s well-being [38].

Another limitation of the study is the absence of a psychological baseline in children anterior to the study, which makes the analysis of stress level variation complex.

Further studies should be performed to better analyze relevant population subtypes within the overall clinical population, such as teenagers and psychopathological subgroups.

## 5. Conclusions

The COVID-19 pandemic has confronted in an unprecedented way the whole population to a suspension of usual human activities. Concerning children’s activities, schools have been closed, with little insight into the potential consequences. For the government, the persistent challenge was to balance the health risks of the disease with the psycho-social risks associated with the protective measures.

Our study allows better identification of the children’s psychological experience during the lockdown. We chose to conduct our study without assuming the direction of the changes we were looking for (increase or decrease in stress levels). Our main results concern the changes in the level of stress experienced by the children: 29% reported a higher level of stress during the lockdown, 34% reported a lower level of stress, and 37% reported no change in stress compared to the usual situation before COVID-19. Our work also allows us to identify certain risk factors associated with an increase or decrease in stress levels, in particular factors related to school (academic pressure and school climate). By highlighting a significant proportion of children for whom lockdown provided immediate relief, our study warns of a possible subsequent risk of increased difficulty in re-exposure to the usual stressors at the time of deconfinement. The reduction in stress experienced by some children during lockdown may, paradoxically, be a risk factor for psychological difficulties in the medium or long term. This work could serve as a warning about the high proportion of children who experience high-stress levels during usual periods, especially related to school attendance. It also highlights the importance of effective and intra-familial support in times of crisis as a key protective factor.

## Figures and Tables

**Table 1:** Univariate and multivariate analysis.

Compared to “Stressed as Usual” (S0)	Decreased Stress Level (S-) N = 2401				Increased Stress Level (S+) N = 2028			
	Univariate Analysis		Multivariate Analysis		Univariate Analysis		Multivariate Analysis	
	Odds-Ratio (95% CI)	*p*-Value	Odds-Ratio (95% CI)	*p*-Value	Odds–Ratio (95% CI)	*p*-Value	Odds-Ratio (95% CI)	*p*-Value
Population Characteristics								
GENDER								
MALE	REFERENCE		REFERENCE		REFERENCE			
FEMALE	1.26 (1.13; 1.41)	<0.001	1.23 (1.08; 1.40)	0.002	1.40 (1.25; 1.57)	<0.001		
ACADEMIC LEVEL								
PRIMARY SCHOOL	REFERENCE				REFERENCE		REFERENCE	
MIDDLE SCHOOL	1.46 (1.29; 1.65)	<0.001			0.90 (0.79; 1.01)	0.089		
HIGH SCHOOL	1.61 (1.36; 1.90)	<0.001			0.96 (0.80; 1.14)	0.614	0.70 (0.54; 0.89)	0.005
LIFESTYLE								
RURAL	REFERENCE				REFERENCE			
URBAN	1.04 (0.92; 1.18)	0.528			1.31 (0.99; 1.29)	0.070		
PARENT 1: SOCIOPROFESSIONAL CATEGORY								
NO PROFESSIONAL ACTIVITY. RETIRED	REFERENCE				REFERENCE			
CRAFTSMEN. TRADERS. FARMERS. AND ENTREPRENEURS	1.15 (0.83; 1.60)	0.396			0.72 (0.53; 1.01)	0.059		
EXECUTIVES AND HIGHER INTELLECTUAL PROFESSIONS	1.12 (0.83; 1.52)	0.453			0.66 (0.49; 0.89)	0.007		
EMPLOYEES AND WORKERS	1.15 (0.86; 1.53)	0.339			0.88 (0.67; 1.16)	0.362		
INTERMEDIARY PROFESSION	1.07 (0.81; 1.43)	0.624			0.72 (0.54; 0.95)	0.019		
PARENT 2: SOCIOPROFESSIONAL CATEGORY								
NO PROFESSIONAL ACTIVITY. RETIRED	REFERENCE				REFERENCE			
CRAFTSMEN. TRADERS. FARMERS AND ENTREPRENEURS	1.05 (0.78; 1.42)	0.682			0.76 (0.55; 1.05)	0.099		
EXECUTIVES AND HIGHER INTELLECTUAL PROFESSIONS	0.90 (0.70; 1.5)	0.586			0.69 (0.54; 0.89)	0.004		
EMPLOYEES AND WORKERS	1.00 (0.78; 1.18)	0.869			0.82 (0.66; 1.01)	0.059		
INTERMEDIARY PROFESSION	1.01 (0.82; 1.26)	0.617			0.83 (0.67; 1.04)	0.103		
CHILD REGULARLY FOLLOWED by a MENTAL HEALTHCARE PROFESSIONAL								
NO	REFERENCE				REFERENCE			
YES	1.58 (1.27; 1.98)	<0.001			2.12 (1.70; 2.65)	<0.001		
LOCKDOWN CONDITIONS								
LIVING with a SIBLING								
NO	REFERENCE				REFERENCE			
YES	1.00 (0.89; 1.14)	0.899			0.91 (0.81; 1.04)	0.162		
LIVING with a PET								
NO	REFERENCE				REFERENCE			
YES	1.15 (1.03; 1.29)	0.014			1.05 (0.93; 1.18)	0.410		
ACTIVITIES								
OUTDOOR ACTIVITIES								
NO	REFERENCE				REFERENCE			
YES	0.95 (0.83; 1.08)	0.426			0.73 (0.64; 0.83)	<0.001		
PLAYING with PET								
NO	REFERENCE				REFERENCE			
YES	1.20 (1.07; 1.35)	0.001			1.06 (0.94; 1.19)	0.341		
GARDENING								
NO	REFERENCE				REFERENCE			
YES	0.59 (0.85; 1.07)	0.431			0.88 (0.77; 0.99)	0.039		
COOKING								
NO	REFERENCE				REFERENCE			
YES	1.12 (1.00; 1.25)	0.061			1.11 (0.99; 1.26)	0.073		
BOARD GAMES								
NO	REFERENCE				REFERENCE			
YES	0.88 (0.79; 0.98)	0.021			0.96 (0.86; 1.08)	0.519		
READING								
NO	REFERENCE				REFERENCE			
YES	0.87 (0.78; 0.97)	0.012			0.97 (0.87; 1.09)	0.661		
DRAWING								
NO	REFERENCE				REFERENCE			
YES	0.91 (0.81; 1.01)	0.084			1.05 (0.94; 1.18)	0.376		
VIDEO GAMES								
NO	REFERENCE				REFERENCE			
YES	0.87 (0.78; 0.98)	0.021			0.84 (0.74; 0.94)	0.004		
MUSIC PRACTICE								
NO	REFERENCE				REFERENCE			
YES	1.03 (0.87; 1.22)	0.706			1.00 (0.84; 1.20)	0.97		
TALKING TO FRIENDS								
NO	REFERENCE				REFERENCE			
YES	1.2 (1.00; 1.25)	0.047			0.96 (0.85; 1.07)	0.433		
SCREEN TIME for HOMEWORK								
<30 min	REFERENCE		REFERENCE		REFERENCE		REFERENCE	
30–60 min	1.04 (0.849; 1.28)	0.704			1.03 (0.84; 1.27)	0.781		
1–2 h	1.23 (1.02; 1.49)	0.027			1.12 (0.92; 1.36)	0.261		
2–4 h	1.49 (1.23; 1.79)	<0.001	1.34 (1.08; 1.67)	0.008	1.27 (1.05; 1.54)	0.016		
>4 h	1.67 (1.34; 2.07)	<0.001			1.82 (1.46; 2.27)	<0.001	1.58 (1.18; 2.12)	0.002
SCREEN TIME for LEISURE								
<30 min	REFERENCE				REFERENCE			
30–60 min	0.98 (0.73; 1.32)	0.89			0.89 (0.66; 1.21)	0.461		
1–2 h	0.97 (0.74; 1.28)	0.84			1.00 (0.75; 1.32)	0.994		
2–4 h	1.04 (0.79; 1.36)	0.797			0.90 (0.68; 1.19)	0.462		
>4 h	1.12 (0.84; 1.48)	0.444			0.88 (0.66; 1.17)	0.378		
SLEEPING PATTERN								
AS USUAL	REFERENCE				REFERENCE			
LITTLE ALTERED	1.05 (0.89; 1.24)	0.575			1.16 (0.97; 1.39)	0.104		
VERY MUCH ALTERED	1.34 (1.13; 1.60)	0.001			1.47 (1.22; 1.77)	<0.001		
NEGATIVE FEELINGS DURING TIME OF LOCKDOWN								
FEAR of GETTING ILL								
NOT AT ALL	REFERENCE		REFERENCE		REFERENCE		REFERENCE	
A LITTLE	1.07 (0.96; 1.21)	0.229			1.88 (1.65; 2.14)	<0.001	1.22 (1.03; 1.45)	0.022
VERY MUCH	1.91 (1.55; 2.36)	<0.001	1.44 (1.12; 1.86)	0.005	7.17 (5.86; 8.78)	<0.001	2.12 (1.61; 2.80)	<0.001
FEAR of a FAMILY MEMBER GETTING ILL								
NOT AT ALL	REFERENCE				REFERENCE		REFERENCE	
A LITTLE	1.05 (0.90; 1.24)	0.534			1.70 (1.38; 2.09)	<0.001		
VERY MUCH	1.56 (1.32; 1.84)	<0.001			4.63 (3.76; 5.70)	<0.001	1.55 (1.18; 2.05)	0.002
WORRY ABOUT LOCKDOWN DURATION								
NOT AT ALL	REFERENCE		REFERENCE		REFERENCE		REFERENCE	
A LITTLE	0.70 (0.62; 0.80)	<0.001	0.85 (0.73; 0.99)	0.042	1.78 (1.53; 2.07)	<0.001	1.22 (1.01; 1.47)	0.038
VERY MUCH	0.74 (0.64; 0.85)	<0.001			3.66 (3.15; 4.25)	<0.001	1.32 (1.09; 1.61)	0.006
WORRY ABOUT ACADEMIC LEARNING								
NOT AT ALL	REFERENCE				REFERENCE		REFERENCE	
A LITTLE	1.07 (0.95; 1.21)	0.246			1.90 (1.65; 2.18)	<0.001	1.47 (1.22; 1.77)	<0.001
VERY MUCH	1.22 (1.03; 1.43)	0.021			5.13 (4.36; 6.03)	<0.001	2.34 (1.84; 2.96)	<0.001
FEELING LESS EFFICIENT for SCHOOL WORK								
NO	REFERENCE				REFERENCE			
YES	0.86 (0.76; 0.98)	0.020			1.87 (1.66; 2.12)	<0.001		
FEELING LESS MOTIVATION for SCHOOL WORK								
NO	REFERENCE				REFERENCE		REFERENCE	
YES	1.02 (0.91; 1.14)	0.769			1.92 (1.70; 2.16)	<0.001	1.56 (1.34; 1.82)	<0.001
SAD to be SEPARATED FROM FRIENDS								
NO	REFERENCE				REFERENCE			
YES	0.76 (0.68; 0.86)	**<0.001**			1.42 (1.24; 1.61)	<0.001		
FEELING of LONELINESS								
NO	REFERENCE		REFERENCE		REFERENCE		REFERENCE	
YES	0.88 (0.75; 1.03)	0.121	0.82 (0.72; 0.94)	0.005	2.41 (2.09; 2.78)	<0.001	1.31 (1.09; 1.58)	0.004
FEELING of BOREDOM								
NO	REFERENCE				REFERENCE			
YES	0.73 (0.65; 0.82)	<0.001			1.37 (1.22; 1.53)	<0.001		
PERCEIVING MORE CONFLICT at HOME								
NO	REFERENCE				REFERENCE		REFERENCE	
YES	1.04 (0.89; 1.22)	0.641			2.00 (1.72; 2.32)	<0.001	1.24 (1.02; 1.51)	0.033
POSITIVE FEELINGS DURING TIME OF LOCKDOWN								
FEELING LESS SCHOOL PRESSURE								
NO	REFERENCE		REFERENCE		REFERENCE		REFERENCE	
YES	2.63 (2.35; 2.95)	<0.001	1.96 (1.71; 2.23)	<0.001	0.80 (0.70; 0.90)	<0.001	0.75 (0.64; 0.88)	0.001
ENJOY MORE FAMILY TIME								
NO	REFERENCE				REFERENCE			
YES	1.08 (0.96; 1.21)	0.233			0.96 (0.85; 1.08)	0.503		
RELIEF of being AWAY from SCHOOL FIGHTS								
NO	REFERENCE		REFERENCE		REFERENCE			
YES	2.04 (1.72; 2.41)	<0.001	1.33 (1.09; 1.62)	0.004	1.85 (1.55; 2.20)	<0.001		
FEELING SUPPORTED by their PARENTS								
NOT AT ALL	REFERENCE				REFERENCE			
A LITTLE	0.93 (0.75; 1.16)	0.517			0.96 (0.76; 1.22)	0.754		
VERY MUCH	0.85 (0.69; 1.05)	0.126			1.06 (0.85; 1.33)	0.624		
WORRIES and EXPECTATIONS about RETURNING to SCHOOL								
WORRY about BEING CONTAMINATED								
NOT AT ALL	REFERENCE				REFERENCE			
A LITTLE	1.33 (1.17; 1.50)	<0.001			1.94 (1.70; 2.20)	<0.001		
VERY MUCH	2.00 (1.66; 2.40)	<0.001			3.37 (2.80; 4.05)	<0.001		
WORRY about BRINGING THE VIRUS to HOME								
NOT AT ALL	REFERENCE				REFERENCE			
A LITTLE	1.19 (1.04; 1.35)	0.1			1.49 (1.30; 1.72)	<0.001		
VERY MUCH	1.72 (1.48; 1.98)	<0.001			2.87 (2.47; 3.34)	<0.001		
FEAR of FALLING BEHIND in LEARNING								
NOT AT ALL	REFERENCE		REFERENCE		REFERENCE		REFERENCE	
A LITTLE	1.22 (1.08; 1.38)	0.001	1.17 (1.01; 1.36)	0.042	1.73 (1.51; 1.98)	<0.001		
VERY MUCH	1.50 (1.27; 1.78)	<0.001	1.31 (1.06; 1.63)	0.013	4.30 (3.66; 5.06)	<0.001	1.34 (1.05; 1.69)	0.016
WORRYING about being SEPARATED from FAMILY								
NOT AT ALL	REFERENCE		REFERENCE		REFERENCE			
A LITTLE	1.29 (1.14; 1.46)	<0.001			1.44 (1.26; 1.63)	<0.001		
VERY MUCH	2.47 (2.06; 2.97)	<0.001	1.76 (1.41; 2.21)	<0.001	2.14 (176; 2.60)	<0.001		
RELIEF about LEAVING the HOUSE								
NOT AT ALL	REFERENCE				REFERENCE			
A LITTLE	0.75 (0.61; 0.93)	0.007			1.16 (0.90; 1.50)	0.259		
VERY MUCH	0.58 (0.48; 0.70)	<0.001			1.57 (1.24; 1.99)	<0.001		
LACKING GUIDANCE in ACADEMIC LEARNING by a PROFESSOR during LOCKDOWN								
NOT AT ALL	REFERENCE				REFERENCE			
A LITTLE	0.92 (0.81; 1.03)	0.157			1.27 (1.12; 1.44)	<0.001		
VERY MUCH	1.05 (0.88; 1.25)	0.607			1.50 (1.25; 1.79)	<0.001		
RELIEF about BEING GUIDED again in LEARNINGS								
NOT AT ALL	REFERENCE		REFERENCE		REFERENCE			
A LITTLE	0.74 (0.64; 0.85)	<0.001	0.75 (0.63; 0.89)	0.001	1.41 (1.18; 1.68)	<0.001		
VERY MUCH	0.69 (0.60; 0.80)	<0.001	0.78 (0.64; 0.95)	0.013	2.69 (2.26; 3.20)	<0.001		
GLAD to RESUME ACTIVITIES								
NOT AT ALL	REFERENCE				REFERENCE			
A LITTLE	0.73 (0.61; 0.88)	0.001			1.37 (1.08; 1.75)	0.011		
VERY MUCH	0.55 (0.46; 0.66)	<0.001			1.82 (1.45; 2.30)	<0.001		
PARENT								
WORRY about CHILD HEALTH								
NOT AT ALL	REFERENCE				REFERENCE			
A LITTLE	1.01 (0.90; 1.14)	0.845			2.16 (1.89; 2.46)	<0.001		
VERY MUCH	1.42 (1.18; 1.72)	<0.001			4.89 (4.09; 5.86)	<0.001		
FEELING that THEIR CHILD was more WITHDRAWN than usual								
NOT AT ALL	REFERENCE		REFERENCE		REFERENCE		REFERENCE	
A LITTLE	1.25 (1.07; 1.46)	0.006			2.40 (2.06; 2.79)	<0.001		
VERY MUCH	1.76 (1.32; 2.35)	<0.001	1.54 (1.10; 2.16)	0.012	4.48 (3.44; 5.85)	<0.001	1.67 (1.17; 2.38)	0.005
FEELING that THEIR CHILD was more DISRUPTIVE than usual								
NOT AT ALL	REFERENCE				REFERENCE		REFERENCE	
A LITTLE	0.87 (0.75; 1.00)	0.051			1.41 (1.22; 1.62)	<0.001		
VERY MUCH	1.06 (0.79; 1.41)	0.698			4.03 (3.16; 5.15)	<0.001	1.39 (1.01; 1.90)	0.043
FEELING that THEIR CHILD was more WORRIED than usual								
NOT AT ALL	REFERENCE				REFERENCE		REFERENCE	
A LITTLE	0.99 (0.87; 1.12)	0.881			4.03 (3.53; 4.62)	<0.001	2.54 (2.16; 2.97)	<0.001
VERY MUCH	1.59 (1.18; 2.15)	0.002			18.82 (14.53; 24.38)	<0.001	5.89 (4.31; 8.06)	<0.001
FEELING that THEIR CHILD was SADDER than usual								
NOT AT ALL	REFERENCE				REFERENCE		REFERENCE	
A LITTLE	0.91 (0.78; 1.05)	0.177			2.88 (2.51; 3.31)	<0.001	1.21 (1.01; 1.45)	0.041
VERY MUCH	1.13 (0.83; 1.53)	0.434			7.21 (5.60; 9.28)	<0.001		
FEELING that THEIR CHILD was more SUFFERING than usual								
NOT AT ALL	REFERENCE				REFERENCE		REFERENCE	
A LITTLE	0.76 (0.67; 0.85)	<0.001			2.78 (2.44; 3.16)	<0.001	1.54 (1.30; 1.81)	<0.001
VERY MUCH	1.09 (0.84; 1.42)	0.533			9.12 (7.26; 11.44)	<0.001	2.13 (1.55; 2.93)	<0.001
FEELING that THEIR CHILD was HAPPIER than usual								
NOT AT ALL	REFERENCE				REFERENCE			
A LITTLE	1.96 (1.72; 2.23)	<0.001			0.84 (0.74; 0.96)	0.011		
VERY MUCH	4.25 (3.63; 4.96)	<0.001			0.76 (0.63; 0.92)	0.005		
FEELING that THEIR CHILD was LESS STRESSED than usual								
NOT AT ALL	REFERENCE		REFERENCE		REFERENCE		REFERENCE	
A LITTLE	3.24 (2.80; 3.74)	<0.001	2.90 (2.49; 3.38)	<0.001	0.91 (0.80; 1.04)	0.159	0.82 (0.70; 0.97)	0.019
VERY MUCH	8.94 (7.63; 10.47)	<0.001	6.26 (5.27; 7.45)	<0.001	1.01 (0.86; 1.20)	0.871		
EMOTIONAL SCORE								
/	REFERENCE				REFERENCE		REFERENCE	
PER POINT	1.12 (1.09; 1.16)	<0.001			1.30 (1.26; 1.34)	<0.001	1.10 (1.06; 1.15)	<0.001

## Data Availability

The complete descriptive data presented in this study are available in Supplementary Materials. The raw data are available on request from the corresponding author.

## Supplementary Materials

The following supporting information can be downloaded at: https://www.mdpi.com/article/10.3390/ijerph20054667/s1.
