# Supplementary material for: Factors Associated with Increased or Decreased Stress Level in French Children during the First COVID-19 Lockdown
Source: Int J Environ Res Public Health. 2023 Mar 6;20(5):4667. doi: 10.3390/ijerph20054667 (PMC10001992; doi:10.3390/ijerph20054667)
Supplement: Supplementary file 1 [file ijerph-20-04667-s001.zip › ijerph-2188221-supplementary.pdf]

Bonjour,

Merci beaucoup d'avoir accepté de participer à ce questionnaire !

Comme tu le sais, le Président de la République a annoncé la fermeture de toutes les écoles à partir du lundi 16 mars. C'était une mesure importante pour limiter le risque de contagion du coronavirus. Depuis, tu continues pourtant à recevoir des enseignements, et tes professeurs se sont organisés pour que l'apprentissage continue malgré le confinement.

Pendant ces 2 mois qui n'étaient pas comme d'habitude, il y a certainement des choses qui t'ont inquiété(e), et d'autres que tu as apprécié.

Le Premier Ministre a maintenant annoncé la reprise progressive de l'école à partir du lundi 11 mai. De la même manière, il y a certainement des choses qui t'inquiètent et d'autres qui te font plaisir autour de cette reprise scolaire.

Dans ce questionnaire, ce sont ces différents aspects de tes ressentis que nous cherchons à mieux identifier, en lien avec ta personnalité et ton contexte de vie.

Le questionnaire que tu vas passer te prendra environ 10 ou 15 minutes. La première partie des questions s'adresse à toi, tandis que la seconde pourra être remplie par tes parents. Il s'agit d'un questionnaire anonymisé, si bien que tes réponses ne peuvent pas être connues par tes professeurs ni par d'autres personnes.

A la fin du questionnaire nous te donnerons quelques conseils sur les impacts psychologiques de l'épidémie, dont tu pourras également discuter avec tes parents.

Bon questionnaire !

NB : Ce questionnaire est réalisé par l'équipe du service de psychopathologie de l'HFME (Pr Fourneret) et par le Centre Régional de Psychotraumatisme de l'hôpital Edouard Herriot (Dr Prieto). Pour toute question au sujet de ce questionnaire, toi ou tes parents pouvez écrire à l'adresse suivante : pauline.espi@chu-lyon.fr

Partie A: Accord parental

A1. **En cliquant sur OUI, vous acceptez la participation de votre enfant au questionnaire.**

Les données sont anonymisées et stockées sur un serveur sécurisé, si bien qu'il n'est pas possible de remonter à votre identité.

- Oui ☐
- Non ☐

Partie B: Données socio-démographiques générales

B1. **Es-tu une fille ou un garçon ?**

- Fille ☐
- Garçon ☐

B2. **Dans quelle classe es-tu ?**

- CP ☐
- CE1 ☐
- CE2 ☐
- CM1 ☐
- CM2 ☐
- 6ème ☐
- 5ème ☐
- 4ème ☐
- 3ème ☐
- Seconde ☐
- Première ☐
- Terminale ☐

B3. **Dans quel type d'établissement scolaire es-tu ?**

- Etablissement public ☐
- Etablissement privé ☐

B4. **Dans quelle ville habites-tu ?**

B5. **Quelle est la profession de tes parents ?**

Partie C: Contexte de vie

C1. **Quels membres de ta famille étaient à la maison avec toi pendant le confinement ?**

- Mère ☐
- Père ☐
- Belle-mère ou beau-père ☐
- Frère(s) ou soeur(s) ☐
- Grands-parents ☐
- Animal de compagnie ☐
- Autre ☐

C2. **Autre :**

C3. **Dans quel logement vivais-tu pendant le confinement ?**

- Maison avec jardin ☐
- Maison sans jardin ☐
- Appartement avec balcon ou terrasse ou cour ☐
- Appartement sans balcon ou terrasse ou cour ☐
- Avec une pièce pour toi seul(e) ☐
- Sans pièce pour toi seul(e) ☐

C4. **Que faisais-tu comme activités pendant le confinement, en plus de tes devoirs ?**

- Activité physique ou jeux à l'extérieur ☐

Jouer avec les animaux ☐

Jardinage ☐

Cuisine ☐

Jeux de société ☐

Lecture ☐

Dessiner ☐

Jeux vidéos ☐

Pratique d'un instrument de musique ☐

Discussion avec tes amis ☐

Autre ☐

C5. Autre :

C6. Combien de temps environ passais-tu devant les écrans par jour (téléphone, TV, ordinateur) ?

	Moins de 30 minutes	Entre 30 minutes et 1h	Entre 1h et 2h	Entre 2h et 4h	Plus de 4h
Pour tes devoirs	☐	☐	☐	☐	☐
Pour tes loisirs	☐	☐	☐	☐	☐

C7. Est-ce que les horaires de ton sommeil et de tes repas étaient modifiés pendant le confinement ?

	Similaires à d'habitude	Un peu modifiés	Beaucoup modifiés	Complètem ent irréguliers
Horaires de coucher - lever	☐	☐	☐	☐
Horaires des repas	☐	☐	☐	☐

Partie D: Scolarité pendant le confinement

D1. **Est-ce que tu t'es senti plus stressé ou moins stressé que d'habitude pendant le confinement ?**

- Beaucoup moins stressé ☐
- Un peu moins stressé ☐
- Pareil que d'habitude ☐
- Un peu plus stressé ☐
- Beaucoup plus stressé ☐

D2. **Est-ce que tu avais des inquiétudes en lien avec l'épidémie de coronavirus ?**

	Pas du tout	Un peu	Beaucoup	Enorméme nt
J'étais inquiet de tomber malade	☐	☐	☐	☐
J'étais inquiet que des membres ma famille tombent malades	☐	☐	☐	☐
J'étais inquiet que le confinement dure longtemps	☐	☐	☐	☐
J'étais inquiet pour mes apprentissages scolaires	☐	☐	☐	☐

D3. **Est-ce qu'il y a des choses que tu as apprécié pendant le confinement ?**

- J'ai eu le sentiment d'apprendre à travailler davantage tout(e) seul(e) ☐
- J'ai eu le sentiment de mieux travailler car je m'organisais comme je voulais ☐
- J'ai ressenti moins de pression en lien avec mes résultats scolaires ☐
- J'ai bien aimé passer plus de temps avec ma famille ☐
- J'étais soulagé(e) d'être moins confronté(e) aux bagarres et moqueries avec les autres élèves ☐
- Je me suis sentie apaisé(e) d'être en dehors du groupe des élèves ☐
- Autre ☐

D4. **Autre :**

D5. **Est-ce qu'il y a des choses que tu n'as pas apprécié pendant le confinement ?**

- J'ai eu le sentiment de moins bien travailler qu'à l'école ☐

J'ai eu le sentiment de ne pas réussir à me motiver pour mon travail scolaire ☐

J'étais triste de ne plus voir mes amis ☐

Je me suis sentie seul(e) ☐

J'ai eu l'impression qu'il y avait davantage de tensions et de disputes à la maison ☐

Je me suis ennuyé(e) ☐

Autre ☐

D6. **Autre :**

D7. **As-tu eu le sentiment que tes parents t'aidaient et t'accompagnaient dans tes travaux scolaires pendant le confinement ?**

Pas du tout ☐

Un peu ☐

Beaucoup ☐

Enormément ☐

Partie E: Reprise scolaire

E1. **As-tu envie de retourner à l'école lorsque ton établissement va ré-ouvrir (ou s'il a déjà ré-ouvert) ?**

Oui ☐

Non ☐

Avec certaines conditions ☐

E2. **Quand tu penses à la reprise de l'école, est-ce que tu as des inquiétudes ?**

	Pas du tout	Un peu	Beaucoup	Enorméme nt
Je suis inquiet d'être contaminé par le coronavirus	☐	☐	☐	☐

	Pas du tout	Un peu	Beaucoup	Enorméme nt
Je suis inquiet de devoir faire les gestes barrières	☐	☐	☐	☐
Je suis inquiet de ramener le virus à la maison	☐	☐	☐	☐
Je suis inquiet d'avoir pris du retard sur mes apprentissages	☐	☐	☐	☐
Je suis triste de devoir me séparer de nouveau de ma famille	☐	☐	☐	☐

E3. Quand tu penses à la reprise de l'école, est-ce qu'il y a des choses qui te font plaisir ?

	Pas du tout	Un peu	Beaucoup	Enorméme nt
Je suis content(e) de retrouver mes amis	☐	☐	☐	☐
Je suis content(e) de retrouver mes professeurs	☐	☐	☐	☐
Je suis soulagé(e) de pouvoir sortir de la maison et de pouvoir bouger	☐	☐	☐	☐
Je suis soulagé(e) de m'éloigner de ma famille pendant la journée	☐	☐	☐	☐
Je suis soulagée d'être de nouveau guidé(e) dans mon travail scolaire	☐	☐	☐	☐
Je suis content(e) de reprendre des activités plus variées	☐	☐	☐	☐

E4. Es-tu retourné au moins une fois à l'école depuis le 11 mai ?

Oui ☐

Non ☐

E5. Qu'est-ce que tu ressens à propos des gestes barrières qui sont mis en place dans ton école ?

	Pas du tout	Un peu	Beaucoup	Enorméme nt
Quand ils portent des masques, les adultes (professeurs, surveillants) me font peur	☐	☐	☐	☐
Je suis triste de ne plus pouvoir toucher mes camarades	☐	☐	☐	☐
Je me sens seul dans la cour de récréation car je n'ai pas droit de m'approcher des autres élèves	☐	☐	☐	☐
J'ai peur que les autres (camarades ou professeurs) s'approchent de moi	☐	☐	☐	☐
Je me sens inquiet en permanence quand je suis à l'école	☐	☐	☐	☐
Je me sens rassuré par ces gestes-barrières car ils luttent contre l'épidémie	☐	☐	☐	☐
J'ai le sentiment de réussir à faire correctement les gestes-barrières	☐	☐	☐	☐

Partie F: Ton tempérament

F1. Voici plusieurs propositions qui cherchent à mieux préciser ton tempérament. Essaye d'indiquer pour chaque proposition si, pour toi, elle est plutôt vraie ou plutôt fausse.

	Totalement faux	Plutôt faux	Plutôt vrai	Totalement vrai
Je suis plutôt timide	☐	☐	☐	☐
J'ai plein d'énergie	☐	☐	☐	☐
Je pleure facilement	☐	☐	☐	☐
Je suis à l'aise avec des gens que je ne connais pas	☐	☐	☐	☐
J'aime beaucoup être en groupe	☐	☐	☐	☐
Je préfère les jeux calmes, sans mouvements, aux activités physiques	☐	☐	☐	☐
Je suis facilement énervé(e)	☐	☐	☐	☐
Je cours du matin au soir	☐	☐	☐	☐
Je suis plutôt sensible	☐	☐	☐	☐

Partie G: Questions pour tes parents

Voici maintenant une série de questions pour tes parents, avant la fin du questionnaire.

G1. Quel était votre niveau d'inquiétude en lien avec l'épidémie pendant le confinement ?

	Pas du tout	Un peu	Beaucoup	Enorméme nt
Inquiétude d'être contaminé et de tomber malade	☐	☐	☐	☐
Inquiétude pour vos proches	☐	☐	☐	☐
Inquiétude pour la scolarité de votre enfant	☐	☐	☐	☐
Nervosité accrue en lien avec le confinement	☐	☐	☐	☐
Manque de confiance dans les autorités publiques pour les prises de décision en lien avec l'épidémie	☐	☐	☐	☐

G2. A la perspective de la reprise scolaire, ressentez-vous des inquiétudes ?

	Pas du tout	Un peu	Beaucoup	Enorméme nt
Inquiétude que votre enfant soit contaminé	☐	☐	☐	☐

	Pas du tout	Un peu	Beaucoup	Enorméme nt
Inquiétude que votre enfant ramène le virus à la maison	☐	☐	☐	☐
Inquiétude que les gestes barrières ne soient pas respectés	☐	☐	☐	☐
Inquiétude logistique pour votre organisation (trajets, reprise du travail, etc)	☐	☐	☐	☐
Inquiétude d'un retard dans les apprentissages de votre enfant pendant le confinement	☐	☐	☐	☐

G3. Votre enfant est-il suivi régulièrement par un psychologue, un pédopsychiatre, ou un professionnel de l'enfance ?

Oui ☐

Non ☐

G4. Avez-vous été particulièrement préoccupé par la santé psychologique de votre enfant pendant le confinement ?

Pas du tout ☐

Un peu ☐

Beaucoup ☐

Enormément ☐

G5. Auriez-vous adressé votre enfant à un professionnel de la santé mentale s'il en avait eu besoin pendant le confinement ?

	Sans hésiter	Eventuelle ment	En dernier recours uniquement
Médecin ou professionnel en libéral	☐	☐	☐
Centre médico-psychologique (CMP) ou autre structure ambulatoire de soins psychiques	☐	☐	☐
Urgences pédiatriques de l'hôpital	☐	☐	☐

G6. Votre enfant a répondu à plusieurs questions sur ses ressentis psychologiques pendant le confinement. De votre côté, qu'avez vous perçu de ses comportements et réactions ?

	Pas du tout	Un peu	Beaucoup	Enorméme nt
J'ai eu l'impression que mon enfant était plus renfermé(e) sur elle/lui-même	☐	☐	☐	☐
J'ai trouvé que mon enfant était plus turbulent que d'habitude	☐	☐	☐	☐
Mon enfant semblait plus inquiet que d'habitude	☐	☐	☐	☐
Mon enfant semblait plus triste que d'habitude	☐	☐	☐	☐
J'ai eu l'impression que mon enfant a souffert du confinement	☐	☐	☐	☐
Mon enfant semblait plus épanoui pendant le confinement	☐	☐	☐	☐
Mon enfant semblait moins stressé que lorsqu'il va à l'école	☐	☐	☐	☐

Merci beaucoup d'avoir participé à ce questionnaire ! Tes réponses sont très précieuses.

Voici quelques conseils maintenant que nous pouvons te donner :

Le confinement peut constituer une source de stress important pour tous les enfants, puisque les repères habituels sont modifiés (ton quotidien change, tu ne vois plus les mêmes personnes, tu ne fais plus les mêmes activités).

Nous te conseillons donc de poser toutes les questions qui t'inquiètent aux adultes autour de toi (tes parents, tes professeurs) ; il est important que tu ne restes pas seul avec tes angoisses. Nous te conseillons aussi de ne pas trop regarder les médias, qui te maintiennent exposé en permanence au stress lié à l'épidémie.

Tu peux trouver d'autres conseils sur le site ci-dessous qui comporte beaucoup de documents ressources :

http://cn2r.fr/wp-content/uploads/2020/03/Que-dire-aux-enfants.pdf

Tu peux aussi montrer ces fiches à tes parents.

Cette petite BD explicative est également un bon moyen de t'informer sur le coronavirus et ses impacts psychologiques :

http://cn2r.fr/wp-content/uploads/2020/03/Le-Coronavirus-explique%CC%81-par-mon-pe%CC%81dopsychiatre.compressed.pdf

Enfin, en cas de <u>détresse psychologique</u> (si tu n'arrives plus à gérer tes angoisses ou ta tristesse, que tu ne dors plus, que tu penses toute la journée à l'épidémie, que tes activités habituelles ne te font plus du tout plaisir, ou encore que tu as des réactions incontrôlées sans comprendre pourquoi), il est essentiel que tu puisses rencontrer un professionnel de la santé mentale.

Tu peux te tourner vers l'infirmière scolaire de ton école, ou tes parents peuvent téléphoner au Centre Médico-Psychologique de la ville où tu habites.

En cas de <u>détresse psychologique intense</u> (si tu as des idées noires, ou que tu fais une crise d'angoisse incontrôlable), il faut que tu demandes à tes proches de t'emmener aux urgences de l'hôpital le plus proche, ou appeler le 15.

	**N = 7218 (%)**
**Gender**	
• Female	3774 (52.3)
• Male	3339 (46.3)
• Missing data	105 (1.5)
**Academic Level** (US equivalent of French system)	
• PrimarySchool	2852 (39.5)
1st Grade	583 (8.1)
2nd Grade	534 (7.4)
3rd Grade	583 (8.1)
4th Grade	621 (8.6)
5th Grade	531 (7.4)
• Middle School	3139 (43.5)
6th Grade	901 (12.5)
7th Grade	859 (11.9)
8th Grade	780 (10.8)
9th Grade (middle school in France)	599 (8.3)
• High School	1112 (15.4)
10th Grade	378 (5.2)
11th Grade	426 (5.9)
12th Grade	308 (4.3)
• Missing data	115 (1.6)
**Level of stress**	
• Higher	2028 (28.1)
• Unchanged	2648 (36.7)
• Lower	2401 (33.3)
• Missing data	141 (2.0)
**Profession Parent 1**	
• No professional activity,	333 (4.6)
• Craftsmen, traders, farmers, and entrepreneurs	633 (8.8)
• Executives and higher intellectual professions	
• Employee / workforce	1046 (14.5)
• Intermediary profession	
	2622 (36.3)
• Missing data	2131 (29.5)
	453 (6.3)
**Profession Parent 2**	
• No professional activity,	669 (9.2)
• Craftsmen, traders, farmers, and entrepreneurs	362 (5.0)
• Executives and higher intellectual professions	
• Employee / workforce	815 (11.3)
• Intermediary profession	
	2553 (35.4)
• Missing data	1852 (25.7)
**Members of the household**	
• Mother	6279 (87.0)
• Father	4827 (66.9)
• Sibling	5052 (70.0)
• Step-father / Step-mother	

• Grand-parents<br>• Missing data	537 (7.4)<br>255 (3.5)<br>@@@
**Type of lodging**<br>• House with exterior<br>• House without exterior<br>• Apartment with Exterior<br>• Apartment without exterior<br>• House<br>• Apartment<br>• With exterior<br>• Without exterior<br>• Missing Data	 5671 (78.6)<br>129 (1.8)<br>965 (13.4)<br>311 (4.3)
**Activities During Lockdown**<br>• Physical activity in exterior<br>◦ Yes<br>◦ No<br>• Gardening<br>◦ Yes<br>◦ No<br>• Playingboardgames<br>◦ Yes<br>◦ No<br>• Cooking<br>◦ Yes<br>◦ No<br>• Reading<br>◦ Yes<br>◦ No<br>• Drawing<br>◦ Yes<br>◦ No<br>• Playing Video Games<br>◦ Yes<br>◦ No<br>• Playing an intrument<br>◦ Yes<br>◦ No<br>• Chatting With Friends<br>◦ Yes<br>◦ No<br><br>**Categories**<br>• Physical activity<br>• Creative or artistic activity<br>• Intellectual Activity<br>• Screen use<br><br>**(No other activity than screen use)**	 <br>5365 (74.3)<br>1853 (25.7)<br><br>2775 (38.4)<br>4971 (68.9)<br><br>3270 (45.3)<br>3948 (54.7)<br><br>4394 (60.9)<br>2824 (39.1)<br><br>3840 (53.2)<br>3378 (46.8)<br><br>3349 (46.4)<br>3869 (53.6)<br><br>4612 (63.9)<br>2606 (36.1)<br><br>818 (11.3)<br>6400 (88.7)<br><br>4018 (55.7)<br>3200 (44.3)<br><br><br>6047 (83.8)<br>3740 (54.8)<br>3846 (53.3)<br>4758 (65.9)<br>107 (1.5)
**Screen Time for school purposes**<br>• < 30 min	 932 (12.9)

• 30 min to 2hrs<br>• 2hrs to 4hrs<br>• > 4 hrs	3096 (42.9)<br>2003 (27.8)<br>1029 (14.3)
**Screen Time for leisure time**<br>- < 30 min<br>- 30 min to 2hrs<br>- 2hrs to 4hrs<br>- > 4 hrs	 339 (4.7)<br>2924 (40.5)<br>2213 (30.7)<br>1536 (21.3)
**Sleeping pattern**<br>• As usual<br>• Little altered<br>• Very much altered<br>• Missing data	 950 (13.2)<br>3769 (52.2)<br>2375 (32.9)<br>124 (1.7)
**Stress confinement**<br>• As usual<br>• Less stressed<br>• More stressed<br>• Missing data	 2648 (36.7)<br>2401 (33.3)<br>2028 (28.1)<br>141 (2.0)
**WORRY ABOUT BECOMING ILL**<br>• Not at all<br>• A little<br>• Very much<br>• Missing data	 3061 (42.4)<br>3078 (42.6)<br>922 (12.8)<br>157 (2.2)
**WORRY ABOUT FAMILY MEMBER BECOMING ILL**<br>• Not at all<br>• A little<br>• Very much<br>• Missing data	 944 (13.1)<br>3105 (43.0)<br>3014 (41.8)<br>155 (2.1)
**WORRY ABOUT LOCKDOWN DURATION**<br>• Not at all<br>• A little<br>• Very much<br>• Missing data	 2641 (36.6)<br>2242 (31.1)<br>2160 (29.9)<br>175 (2.4)
**WORRY ABOUT ACADEMIC LEARNINGS**<br>• Not at all<br>• A little<br>• Very much<br>• Missing data	 2963 (41.1)<br>2614 (36.2)<br>1471 (20.4)<br>170 (2.4)
**LESS EFFICIENT in their SCHOOL WORK**<br>• No<br>• Yes	 5140 (71.2)<br>2078 (28.8)
**LESS MOTIVATED in their SCHOOL WORK**<br>• No<br>• Yes	 4559 (63.2)<br>2659 (36.8)

**SAD to be SEPARATED FROM FRIENDS**	
• No	2344 (32.5)
• Yes	4874 (67.5)
**FEELS LONELY**	
• No	5886 (81.5)
• Yes	1332 (18.5)
**FEELS BORED**	
• No	4322 (59.9)
• Yes	2896 (40.1)
**SENSE MORE TENSION AT HOME**	
• No	5984 (82.9)
• Yes	1234 (17.1)
**FEELLESS ACADEMIC PRESSURE**	
• No	4508 (62.5)
• Yes	2710 (37.5)
**ENJOY MORE FAMILY TIME**	
• No	2419 (33.5)
• Yes	4799 (66.5)
**ENJOY LESS SCHOOL ALTERCATIONS**	
• No	6224 (86.2)
• Yes	994 (13.8)
**FEEL SUPPORTED by their PARENTS**	
• Not at all	552 (7.6)
• A little	2129 (29.5)
• Very much	4396 (60.9)
• Missing data	141 (2.0)
**WORRY about BEING CONTAMINATED**	
• Not at all	3495 (48.4)
• A little	2585 (35.8)
• Very much	966 (13.4)
• Missing data	172 (2.4)
**WORRY about BRINGING THE VIRUS HOME**	
• Not at all	2451 (34.0)
• A little	2606 (36.1)
• Very much	1976 (27.4)
• Missing data	185 (2.6)
**Worry About Falling Behind In School Learning**	
• Not at all	3146 (43.6)
• A little	2531(35.1)
• Very much	1365 (18.9)
• Missing data	176 (2.4)

**WORRY about BEING SEPARATED from FAMILY**<br>• Not at all<br>• A little<br>• Very much<br>• Missing data	 3975 (55.1)<br>2174 (30.1)<br>866 (12.0)<br>203 (2.8)
**RELIEF about LEAVING the HOUSE**<br>• Not at all<br>• A little<br>• Very much<br>• Missing data	 625 (8.7)<br>1701 (23.6)<br>4699 (65.1)<br>193 (2.7)
**RELIEF about GETTING AWAY from FAMILY**<br>• Not at all<br>• A little<br>• Very much<br>• Missing data	 3581 (49.6)<br>2524 (35.0)<br>902 (12.5)<br>211 (2.9)
**RELIEF about BEING GUIDED in their LEARNINGS**<br>• Not at all<br>• A little<br>• Very much<br>• Missing data	 1462 (20.3)<br>2914 (40.4)<br>2637 (36.5)<br>205 (2.8)
**GLAD to RESUME ACTIVITIES**<br>• Not at all<br>• A little<br>• Very much<br>• Missing data	 708 (9.8)<br>2350 (32.6)<br>3973 (55.0)<br>187 (2.6)
**WORRY about CHILD HEALTH**<br>• Not at all<br>• A little<br>• Very much<br>• Missing data	 3454 (47.9)<br>2484 (34.4)<br>1035 (14.3)<br>245 (3.4)
**REPORT CHILD WITHDRAWN**<br>• Not at all<br>• A little<br>• Very much<br>• Missing data	 5317 (73.7)<br>1241 (17.2)<br>413 (5.7)<br>247 (3.4)
**REPORT CHILD having BEHAVIORAL DIFFICULTIES**<br>• Not at all<br>• A little<br>• Very much<br>• Missing data	 5096 (70.6)<br>1426 (19.8)<br>449 (6.2)<br>247 (3.4)
**REPORT CHILD being WORRIED**<br>• Not at all<br>• A little<br>• Very much<br>• Missing data	 3972 (55.0)<br>2345 (32.5)<br>652 (9.0)<br>249 (3.4)

**REPORT CHILD being SAD**	
• Not at all	4838 (67.0)
• A little	1642 (22.7)
• Very much	474 (6.6)
• Missing data	264 (3.7)
**REPORT CHILD SUFFERING**	
• Not at all	3729 (51.7)
• A little	2593 (35.9)
• Very much	644 (8.9)
• Missing data	252 (3.5)
**REPORT CHILD HAPPY**	
• Not at all	3487 (48.3)
• A little	2163 (30.0)
• Very much	1297 (18.0)
• Missing data	271 (3.8)
**REPORT CHILD LESS STRESSED**	
• Not at all	2921 (40.5)
• A little	2254 (31.2)
• Very much	1784 (24.7)
• Missing data	259 (3.6)
**EMOTIONAL SCORE**	
• 0	160 (2.2)
• 1	280 (3.9)
• 2	653 (9.0)
• 3	1053 (14.6)
• 4	1297 (18.0)
• 5	1313 (18.2)
• 6	1037 (14.4)
• 7	641 (8.9)
• 8	405 (5.6)
• 9	190 (2.6)
• Missing data	89 (2.6)
**SOCIABILITY SCORE**	
• 0	66 (0.9)
• 1	203 (2.8)
• 2	426 (5.9)
• 3	844 (11.7)
• 4	1276 (17.7)
• 5	1271 (17.6)
• 6	1284 (17.8)
• 7	942 (13.1)
• 8	480 (6.7)
• 9	228 (3.2)
• Missing data	198 (2.7)
**ACTIVITY SCORE**	
• 0	32 (0.4)
• 1	129 (1.8)

• 2	360 (5.0)
• 3	614 (8.5)
• 4	1085 (15.0)
• 5	1417 (19.6)
• 6	1339 (18.6)
• 7	945 (13.1)
• 8	681 (9.4)
• 9	399 (5.5)
• Missing data	217 (3.0)
